# Cellular reaction gene regulation network for swarm robots with pattern formation maneuvering control

**DOI:** 10.3389/fnbot.2022.950572

**Published:** 2022-10-20

**Authors:** Zhenlong Xiao, Xin Wang, Lin Hong

**Affiliations:** Department of Mechanical and Automation Engineering, Harbin Institute of Technology, Shenzhen, China

**Keywords:** pattern formation, maneuver control, cellular reaction networks, gene regulation networks, morphogen diffusion

## Abstract

Self-organized pattern formation enables swarm robots to interact with local environments to self-organize into intricate structures generated by gene regulatory network (GRN) control methods without global knowledge. Previous studies have reported that it is challenging to maintain pattern formation stability during maneuvering in the environment due to local morphogenetic reaction rules. Motivated by the mechanism of the GRN in *multi-cellular organisms*, we propose a novel cellular reaction gene regulatory network (CR-GRN) for pattern formation maneuvering control. In CR-GRN, a cellular reaction network is creatively proposed to depict the robots, environment, virtual target pattern, and their interaction to generate emergent swarm behavior in multi-robot systems. A novel diffusion equation is proposed to simulate the process of morphogen diffusion among cells to ensure stable adaptive pattern generation. In addition, genes, proteins, and morphogens are used to define the internal and external states of cells and form a feedback regulation network. Simulation experiments are conducted to validate the proposed method. The results show that the CR-GRN can satisfy the requirements of turning curvature and maintain the robot's uniformity based on the proposed algorithm. This proves that robots using the CR-GRN can cooperate more effectively to cope in a complicated environment, and maintain a stable formation during maneuvering.

## 1. Introduction

In multi-cellular organisms, all metabolic processes occur in a complex feedback-controlled metabolic network followed by simple central dogma maintaining homeostasis. The gene regulatory network (GRN) process results in massive complex patterns adapted to evolving ecological environments, such as bacterial colony structures, slime mold networks, and zebra skin. In the recently emerging field of multi-robot systems (MRSs), the principles of the natural GRN were introduced into robotics.

In robotics, it was found that a single robot cannot solve many problems with multiple functions. In contrast, swarm robot technology utilizes a robot swarm composed of low-cost, low-complexity, and high-redundancy individual robots that can make these problems more solvable. Inspired by biological systems, many problems have been solved by using biological heuristic algorithms, such as pattern formation (Spears et al., 2004; Sperati et al., 2011; Spears and Spears, 2012; Rubenstein et al., 2014; Slavkov et al., 2018), objective search (Hayes, 2002; Kantor et al., 2003; Zhang et al., 2014; Yang et al., 2019), collective decision-making (Couzin et al., 2005; Amé et al., 2006; Garnier et al., 2009; Francesca et al., 2012), and unmanned aerial vehicle formation flight (Chung et al., 2018). In particular, considerable attention has been paid to adaptive pattern formation to allow swarm robots to move in a baggy or compact pattern formation, which is an essential function for performing a given task. The proposed algorithms, inspired by biology, for pattern formation are mainly divided into two categories. The first is the macro-behavior-inspired pattern formation algorithms, such as the ant colony algorithm (Dorigo and Gambardella, 1997), artificial bee colony algorithm (Karaboga and Basturk, 2007), particle swarm optimization (Kennedy and Obaiahnahatti, 1995), virtual structure (Lewis and Tan, 1997), and potential field (Gazi, 2006). Traditional bio-inspired algorithms mostly mimic macroscopic creature behaviors or physical phenomena. The other is multi-cell-mechanic-inspired pattern formation algorithms. These include the Morphogen diffusion model, Reaction-diffusion model (Turing, 1952), GRN model (Jin et al., 2012), and Chemotaxis (Eisenbach, 2004). As the GRN provides a promising solution for pattern formation, numerous algorithms based on the GRN principle have been proposed, such as reaction-diffusion (Slavkov et al., 2018) and hierarchical GRN (H-GRN) algorithms (Jin et al., 2012).

However, it is not easy to sustain a predefined pattern when the robots maneuver around the environment (Oh et al., 2017). In the aforementioned methods, only a few features were used to create bio-inspired control systems, and others were dropped, causing these systems to be defective. For example, morphogen diffusion models, which generate patterns by morphogen gradients, were not designed for real-time systems, and it was challenging to maintain the stability of the pattern. Furthermore, biological systems are different from robot systems; thus, subtle analogies between organisms and robots are needed.

In observing natural phenomena, we noticed that the pattern of organisms produced by morphogens is stable over a long period (usually for decades). Organisms that grow in a certain shape usually contain two types of cells, rather than one. One cell type is a relatively rigid structural cell used to form shape constraints, such as bones, skin, or dura. The other type of cells with specific functions is less rigid, and its shape is constrained by structural cells, such as skeletal muscle, brain, and red blood cells (Netter and Colacino, 1989). These different types of cells work together to form a real-time biosystem of cell networks in which cells “react” with each other by regulating genes.

Based on the aforementioned studies and observations, we propose an algorithm called the cellular reaction gene regulatory network (CR-GRN), which mimics the properties of multiple cell types to maintain a rigid pattern structure. The CR-GRN consists of three layers: (1) Layer 1 is morphogenetic diffusion, which is a multi-source static diffusion method based on the Dirac delta function (Murray, 2002). The diffusion of morphogens is considered to provide implicit location information on cell positioning and movement. (2) Layer 2 is cellular reaction layer, which is used to provide the properties of multiple cell types to improve adaptive capacity to environment. (3) Layer 3 is motion control layer, which is a GRN layer driving the robots to target positions generated by layer 1 and layer 2. The main contributions of this paper can be listed as follows.

1. A three layers CR-GRN is proposed to maintain the pattern formation of swarm robots during maneuvering.
2. The algorithm is considered to have stable pattern generation and maintenance ability, adaptive change ability, and limited obstacle avoidance ability.
3. The above problems are verified by designing robot simulations.

The remainder of this paper is organized as follows. In Section 2, we review related studies in the field of pattern formation. In Section 3, the details of the CR-GRN are introduced. Section 4 presents a discussion on the performance of the CR-GRN and its comparison with that of the H-GRN in the trapping target task. Finally, the conclusion of the study is provided in Section 5.

## 2. Related works

Some bio-inspired methods have been proposed for the pattern formation of MRSs over the past few decades. In many research scenarios, robots are arranged in tight or loose formations, which are considered to be the primary function for completing corresponding tasks (Oh et al., 2017). A centralized approach can complete pattern formation in a few exceptional cases, and it is feasible in controlled environments and small scenarios. However, centralized approaches cannot guarantee a swarm's tolerance for error and fragile communication capabilities with a complex external environment. To address these problems, many decentralized formation algorithms have been proposed. These algorithms can be divided into two main categories: (1) collective-behavior-based algorithms and (2) multi-cell-mechanics-based algorithms. In behavior-based algorithms, the leader-follower model, in which the leader robot needs to be assigned, was the first to be identified. In this section, we review the background of these problems from the perspective of bio-inspired methods, including behavior-based and multi-cell-mechanics-based algorithms.

### 2.1. Collective-behavior-based algorithms

#### 2.1.1. Leader-follower

A leader-follower framework was established by Alur et al. (2001), and linear feedback was used to keep followers in line with the leader's movement. Panagou and Kumar (2014) proposed a movement location and control strategy to solve pattern formation in the presence of visual and communication constraints in a known obstacle environment. Obtaining the adaptability of the formation has received much attention in pattern maintenance. Consolini et al. (2008) moved the followers in an arc around the leader and maintained their distance. Yang et al. (2018) proposed a v-shaped formation control method to imitate the formation of wild geese and conducted a large-scale simulation experiment using the stage simulation tool, which showed good expansibility. This type of algorithm depends on leaders' existence, and their formation is relatively rigid; thus, they are difficult to adapt to unknown environments and errors.

#### 2.1.2. Virtual structure models

Virtual structure models were proposed to solve the leader-follower model's problem, where their patterns are too simple, and the robot's formation is treated as a single entity. The target position of the robot is distributed in a fixed structure. Oh et al. proposed a control method for circular formation to track dynamic targets (Oh et al., 2014). Although the virtual structure method overcame the leader-follower model's dependence on the leader-robot, the two models shared some defects.

#### 2.1.3. Potential field

Another important model is the potential field. Robots are constrained to a range under virtual attraction and repulsion using potential field models. Pimenta et al. (2007) imitated the movement of a fluid in an electrostatic field by repelling the robot away from obstacles and attracting them to the target.

### 2.2. Multi-cell-mechanics-based algorithms

#### 2.2.1. Morphogen diffusion and reaction-diffusion

An important study is the simulation of artificial life and mathematical modeling of cellular development (Gierer and Meinhardt, 1972; Ingham, 1988; Jaeger et al., 2004; Isaeva, 2012; Sheth et al., 2012). It is fascinating how simple laws generate complex patterns of organisms. It was found that the formation of biological patterns depends on changes in the concentration of morphogens. The study of morphogen diffusion originated from Turing's work on morphogenesis in 1952 (Turing, 1952). Turing elaborated on how morphogens influence biological patterns by reaction-diffusion. He summarized the reaction-diffusion problem as the migration and reaction changes of morphogens in cells.

The diffusion problem developed further after Turing's work. The way molecules move in microscopic environments began to be explored, and it can be defined as the diffusion process, which is the collection of disordered movement of single particles. The diffusion problem can be simplified into the random walk of particles.

There are no coordinate systems in living organisms, where the cells can locate each other based on the concentration of morphogens. This feature makes these principles applicable to pattern formation in MRSs. Earlier studies were based on simple morphogen diffusion models. For example, Nagpal et al. (2002) and Kondacs (2003), using morphogenesis and geometry, developed a self-organizing algorithm that can generate 2-D graphics.

#### 2.2.2. GRN

Among the current pattern formation methods, the GRN algorithm originating from the Turing reaction-diffusion model has a significant contribution to the formation problem. In addition to designing the general controllers of the collective robot with collective-behavior-based methods, the introduced cell-inspired control system shows good performance (Guo et al., 2011). The robots only obtain the neighbors' information for the local self-organization method. Slavkov divided the robots into two local self-organized groups tracking different morphogen concentrations, U and V, which behave as activators and inhibitors (Slavkov et al., 2018). Although the algorithm can generate certain regular shapes, they are generally uncontrollable by will. Rubenstein et al. (2014) allowed thousands of robots to form predefined shapes. However, their methods have some limitations, including but not limited to the following:

- Several “seed robots” are needed to form the coordinate system.
- There is no interaction with the environment.
- Only a few robots can move at the same time.

Jin tried to solve the target trapping problem with the GRN method (Jin et al., 2012). Based on the study conducted by Guo et al. (2011), Jin proposed a H-GRN method with a two-layer structure, which targets the function of secreting virtual “morphogen molecules” and describes the gradient pattern with the Non-uniform Rational B-Splines (NURBS) model. NURBS is a classical curve description model. Although some improvements have been made in subsequent studies, the applications are still limited. For instance, the NURBS model is replaced by the Radial Basis Implicit Function in the work of Zhang et al. (2018). Some defects of the H-GRN include the following: (1) The maps need to be maintained and updated to record gradient changing of morphogens. (2) The pattern based on the differential equation's diffusion model would be lost over time.

#### 2.2.3. Chemotaxis

Chemotaxis is a mechanism that directs the movement of cells, which release chemicals into their surroundings, and other cells respond by approaching or leaving the environment (Eisenbach, 2004). Chemotaxis can produce complex patterns, but could not get an expected shapes (Bai et al., 2008).

## 3. Problem statement and assumptions

The problem involves a group of robots maneuvering in space and maintaining pattern formation according to the predefined pattern. The coordinates of each robot form a predefined pattern in 2D space. The robots need to maintain the pattern formation adapted to the movement's environment.

When passing through the obstacle area, the robot formation should undergo adaptive deformation. Then, the original formation should be restored as soon as possible. To make the algorithm more applicable, the following constraints for multi-robots are formulated:

- The robots can locate the relative position of obstacles and their companions in the local coordinate system.
- The computing power is relatively low.
- The robot has no other obstacle avoidance algorithm, and the obstacle avoidance ability only depends on the proposed CR-GRN.
- Every individual robot is treated as a particle in simulation.
- In the process of robots motion, the collision problem between the robots is not considered. It is assumed that the robots prevent collisions by additional collision avoidance strategies.

## 4. CR-GRN

This article presents a pattern formation algorithm called the CR-GRN to perform swarm maneuvering in the environment in the expected pattern. Each robot has both an independent and same controller based on the CR-GRN algorithm. According to the hypothesis, the robot can obtain the relative positions of the neighbors and obstacles in the local coordinate system within the perceived range. The proposed CR-GRN consists of three abstraction layers:

1. Morphogenetic diffusion layer;
2. Cellular reaction layer;
3. Movement control layer.

**Figure 1:**
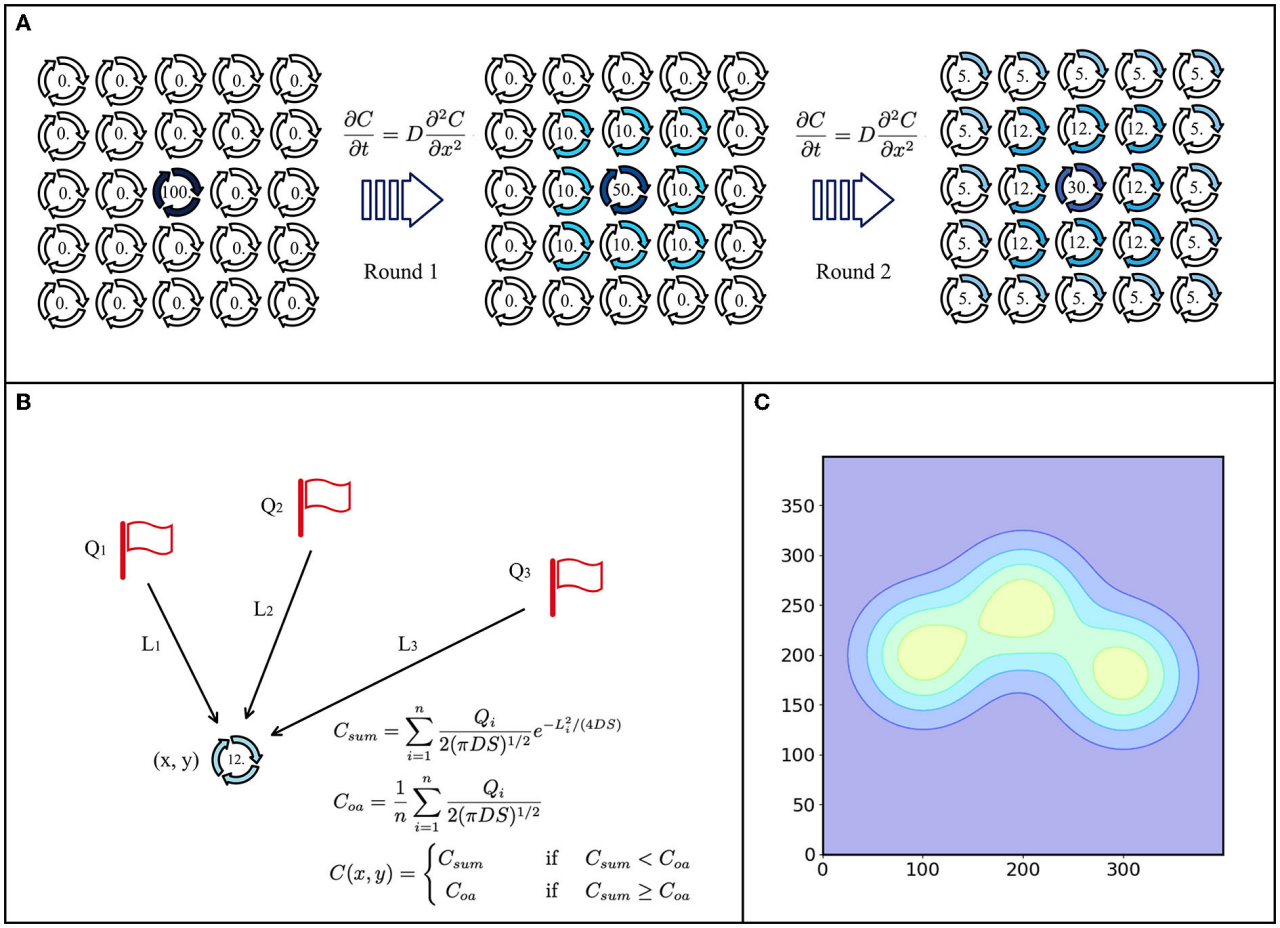
Morphogen diffusion model. **(A)** Dynamic diffusion model. There is an initial concentration at the center, and then, it spreads around each round. **(B)** Static diffusion model. Three points have an initial concentration (Q1, Q2, and Q3) and distances (L1, L2, and L3) to position (x, y), where the concentration can be calculated by using the static diffusion model. **(C)** Diffusion gradient patterns of A and B.

The morphogenetic diffusion layer generates adaptive patterns by morphogenetic diffusions secreted by different cells. Inspired by the Dirac-Delta equation, we propose a static diffusion equation to replace the traditional dynamic diffusion equation based on Fick's diffusion law in the morphogenic diffusion layer. The traditional diffusion equation is limited by the unstable concentration maps updated by the robots and is difficult to be applied in practice, as shown in Figure 1A. The static diffusion equation used in the morphogenetic diffusion layer can generate stable patterns that do not change over time and yield repeatable results, as shown in Figure 1B.

**Figure 2:**
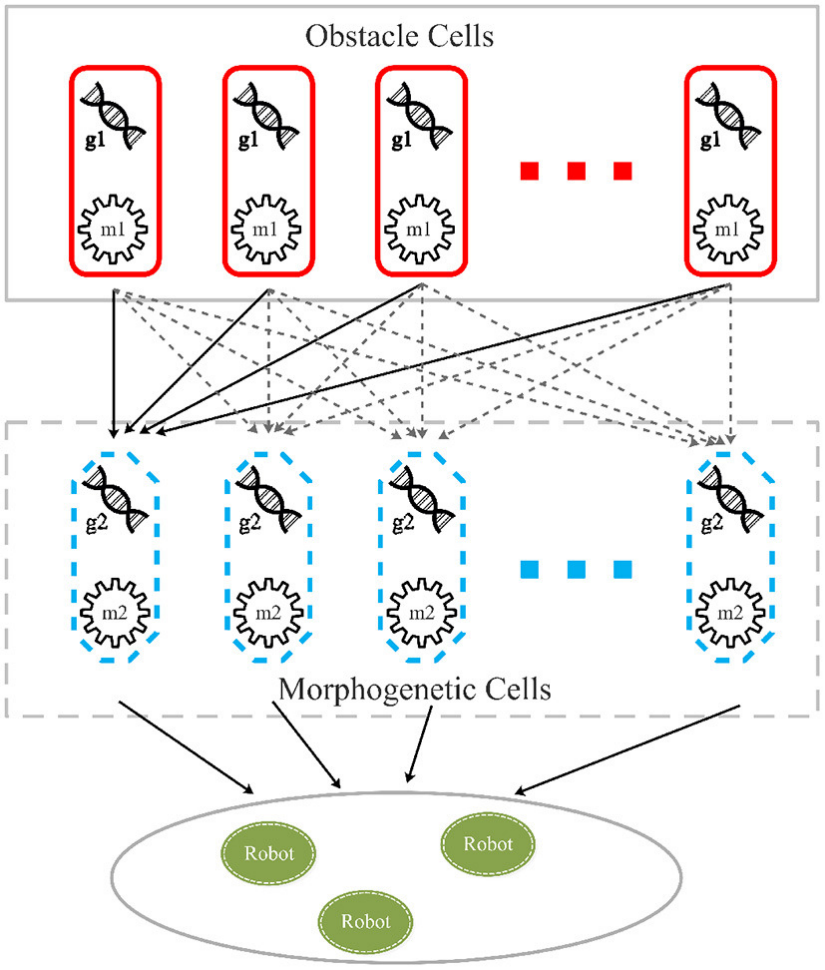
Cellular reaction model. *Obstacle cells* are obstacles represented by a group of cells containing gene g1, which is translated into morphogen m1. *Morphogenetic cells*, which contain gene g2 suppressed by m1, represent a predefined pattern. *Robot cells* represent the motion control layer translating morphogen m2, a protein translated by gene g1, into the NURBS model and guide robots to move to target on the pattern.

The cellular reaction layer provides the reaction mechanism between objects, which corresponds to the feedback regulation mechanism of the robot control system. In this layer, the genes of real and virtual objects represented by cells are regulated by morphogens secreted by cells at the upper level of the environment, and synthetic morphogens regulate the next level, as shown in Figure 2.

In the movement control layer, the modified information of the position is translated and leads robots to target positions by feedback control from the gene regulation network.

### 4.1. Morphogenetic diffusion layer

**Figure 3:**
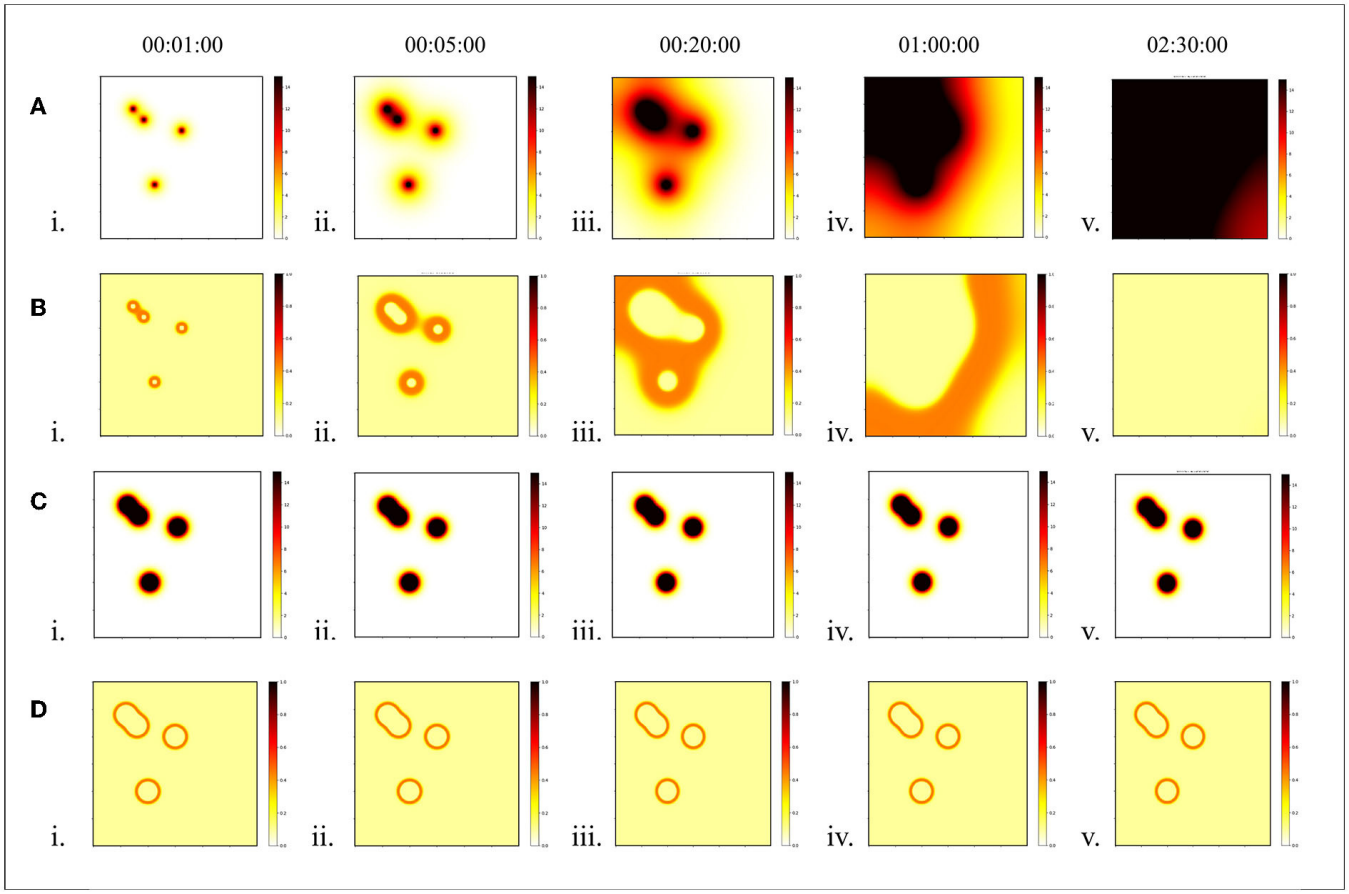
Stability of morphogen diffusion. In this experiment, the stability of the static diffusion method was compared with that of the dynamic diffusion method. Each time interval was set at 1 s. **(A)** Concentration distribution of dynamic diffusion and **(B)** pattern of dynamic diffusion. **(C)** Concentration distribution of static diffusion, and **(D)** pattern of static diffusion.

**Figure 4:**
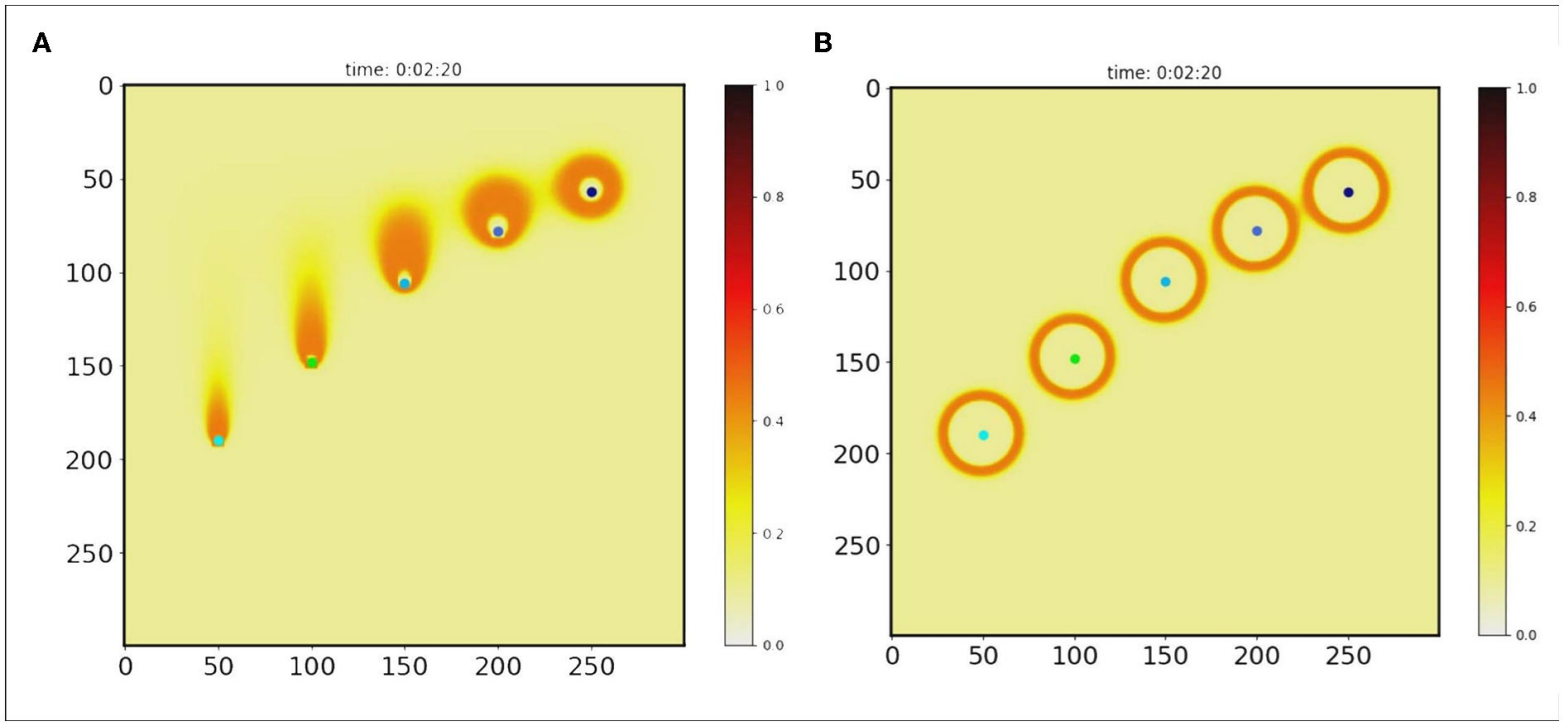
Pattern generation. It is assumed that morphogen is secreted by points (colored points). And each point moved with velocity (V = 1.35, 1.07, 0.78, 0.57, 0.43 cm/s). Two algorithms were used to extract orange rings as patterns. **(A)** H-GRN, **(B)** CR-GRN.

The diffusion pattern of morphogens depends on the gradient of its concentration distribution in space. To form a stable pattern, it is expected that the distribution of morphogen concentrations will remain in one state. However, it is difficult to maintain the stability of the distribution as dynamic methods continue to spread to neighborhoods over time. This defect made the pattern obtained from the GRN unstable, as shown in Figure 3A. Moreover, the pattern will fall apart when it moves a little faster, as shown in Figure 4A. When the speed exceeds 0.57 cm/s, the pattern cannot be maintained stably.

The dynamic method requires concentration maps, which limit the robot's range of movement and require more computing resources to record and update the distribution changes of morphogens in the environment, as shown in Figure 1A. Inspired by the Dirac delta function (Murray, 2002), we propose a static distribution method to replace the differential equation:


(1) $$\begin{array}{l} C\left(L\right)=\frac{Q}{2{\left(\pi DS\right)}^{1/2}}e^{-L^{2}/\left(4DS\right)} \end{array}$$

*Q* denotes the intensity of the diffusion source, such as an obstacle. *C*(*L*) denotes the concentration of location (*x, y*). *L* denotes the distance from the point to the diffusion source. *D* is the diffusion coefficient. *S*, treated as a diffusion state, is the time from the beginning of the diffusion. The morphogen concentration can be calculated by this equation at any time and at any location without waiting for the gradual change of the morphogen. Another problem to be considered in concentration diffusion is the multi-source problem, as shown in Figure 1B. For location (*x, y*), there are *n* × diffusion sources. In the case of single-source diffusion, the concentration of (*x, y*) is *C*(*x, y*). The sum of the concentrations of (*x, y*) for multiple sources is


(2) $$\begin{array}{l} C_{sum}=\overset{n}{\underset{i=1}{\sum }}\frac{Q_{i}}{2{\left(\pi DS\right)}^{1/2}}e^{-L_{i}^{2}/\left(4DS\right)} \end{array}$$

Here, *Q*_*i*_ is the intensity of the *ith* diffusion source. If *L* = 0, the average concentration of diffusion sources is


(3) $$\begin{array}{l} C_{oa}=\frac{1}{n}\overset{n}{\underset{i=1}{\sum }}\frac{Q_{i}}{2{\left(\pi DS\right)}^{1/2}} \end{array}$$

(*x, y*) is the coordinate of any point in the local coordinate system in the environment. Then the actual concentration of (*x, y*) point in multi-sources can be defined as


(4) $$\begin{array}{l} C\left(x,y\right)=\{\begin{array}{cc} C_{sum} & \;\text{if}\;C_{sum}<C_{oa}\\ C_{oa} & \;\text{if}\;C_{sum}\geq C_{oa} \end{array} \end{array}$$

This equation implies that the concentration at any point is *C*_*sum*_, the sum of the concentrations of each diffusion from different sources, but that the sum concentration does not exceed the average of the source concentrations. Therefore, the maximum morphogenetic concentration should be the average of the concentration of the source of morphogenetic secretion *C*_*oa*_. The static and dynamic diffusion methods share the same diffusion gradient pattern, as shown in Figure 1C. As we can see, the pattern (Figures 3C,D and Figure 4B) formed by the static diffusion method (Equation 4) is constant from start to end.

### 4.2. Cellular reaction layer

**Figure 5:**
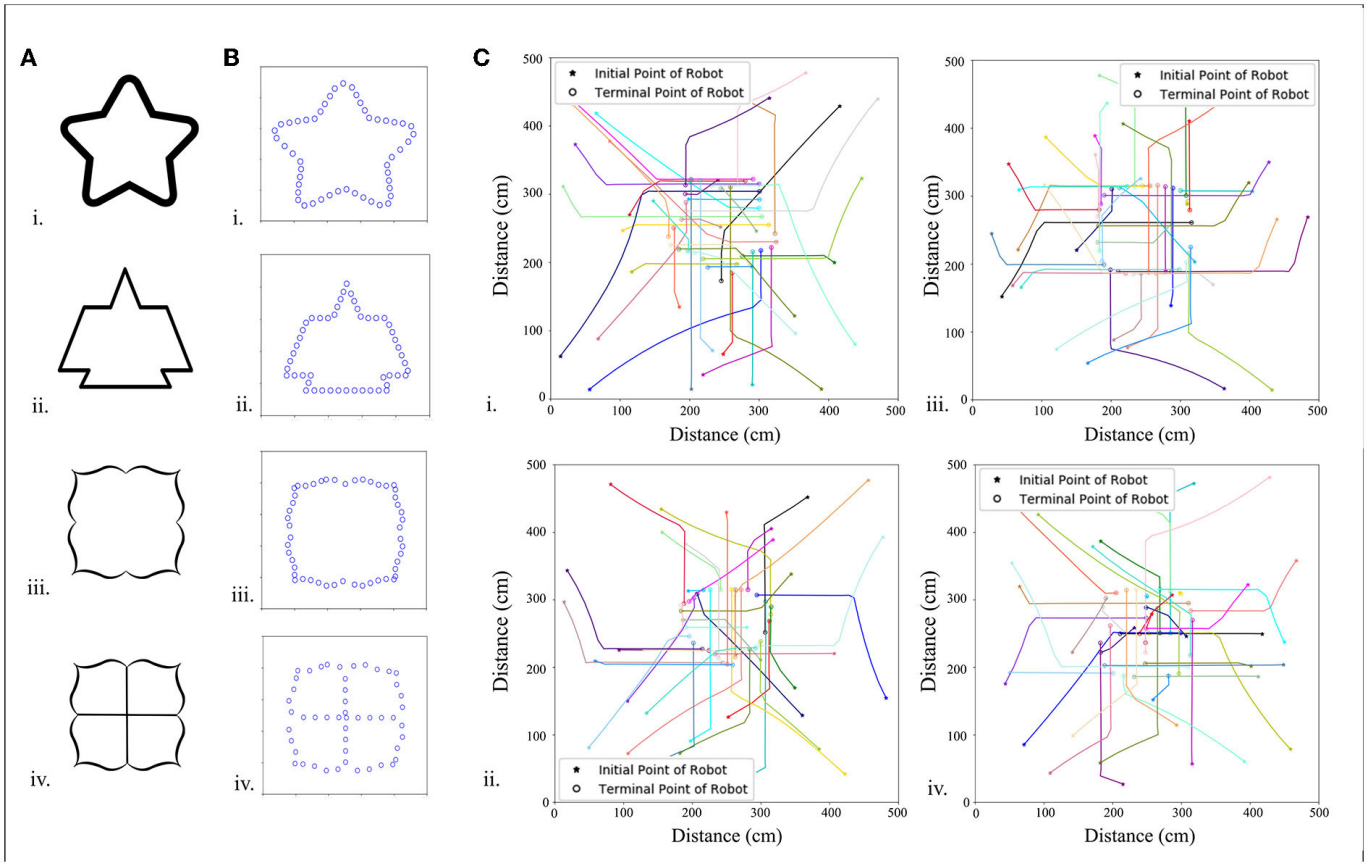
Predefined pattern determined by pictures. **(A)** Images of different patterns. **(B)** Each pattern has 50 morphogenetic cells (blue circles) arranged along the black line, which is translated into the NURBS model, and **(C)** trajectories of 20 robots arranged in patterned formations.

In the CR-GRN model, we innovatively propose the cellular reaction layer. Different objects are represented by different cell types in this layer, such as obstacles represented by obstacle cells expected patterns represented by morphogenetic cells and robots represented by robot cells. Each cell has its own gene and its corresponding morphogen. For example, obstacle cells have gene g1 and corresponding morphogen m1. Morphogens secreted by one cell regulate gene activity in another cell, regulating cellular reactions. For example, morphogen m1 inhibits gene g2 and affects pattern formation. As shown in Figure 3, it is assumed that morphogenetic cells are used to represent the expected pattern obtained from the image (Figure 5A). Morphogenetic cells would interact with obstacles represented by cells to modify the pattern. The expected pattern is converted from a picture into the NURBS model to determine the relative positions of the morphogenic cells lined up along it, as shown in Figure 5B.

Morphogenetic cells that generate morphogens secreted into the environment are a series of virtual cells arranged along the expected pattern obtained by binarization and skeletonization. In addition, 200 points were randomly chosen in the foreground pixels of a skeletonized image as control points to build a NURBS model. Here, it is set that each pattern consists of 50 morphogenetic cells. Because the NURBS model accepts input parameters from 0 to 1, each cell has a NURBS parameter value, *N*_*i*_, *N*_*i*_ = *i*/*cells*_*num* to obtain the position in the pattern, where i denotes the number of the cell. It is hypothesized that the morphogenetic cell has gene *g*_2_ that synthesizes and secretes the morphogenetic protein, *m*_2_. Morphogen *m*_1_ from the environment (obstacle cells) inhibits the gene *g*_2_ and reduces the concentration of *m*_2_, as shown in Figure 2. The morphogen diffusion of obstacle cells is defined by Equation (4).

*C*_*m*1_, the concentration of morphogen *m*_1_ inside obstacle cells, is determined by *K*_*g*1_, the activity factor of gene *g*_1_:


(5) $$\begin{array}{l} C_{m1}=K_{g1}I_{1} \end{array}$$

Because *K*_*g*1_ is not affected by any morphogens, the value of *K*_*g*1_ is 1. *I*_1_ denotes the initial gene activity, *g*_1_. For morphogenetic cells, the secretion of morphogens is affected by the morphogen concentration of obstacle cells, as follows:


(6) $$\begin{array}{l} C_{m2}=K_{g2}I_{2} \end{array}$$

(7) $$\begin{array}{l} K_{g2}=1-Sig\left(C_{m1}\left(T\right),\theta_{1},k_{1}\right) \end{array}$$

where *C*_*m*2_(*x*) denotes the concentration of morphogen *m*_2_ in the morphogenic cells, which is calculated using Equation (4). *K*_*g*2_ denotes the activity factor of gene *g*_2_. *I*_2_ denotes the initial activity of *g*_2_. Sig is the sigmoid equation in which θ and k are parameters.


(8) $$\begin{array}{l} Sig\left(x,z,k\right)=\frac{1}{1+e^{-k\left(x-z\right)}} \end{array}$$

### 4.3. Motion control layer

**Figure 6:**
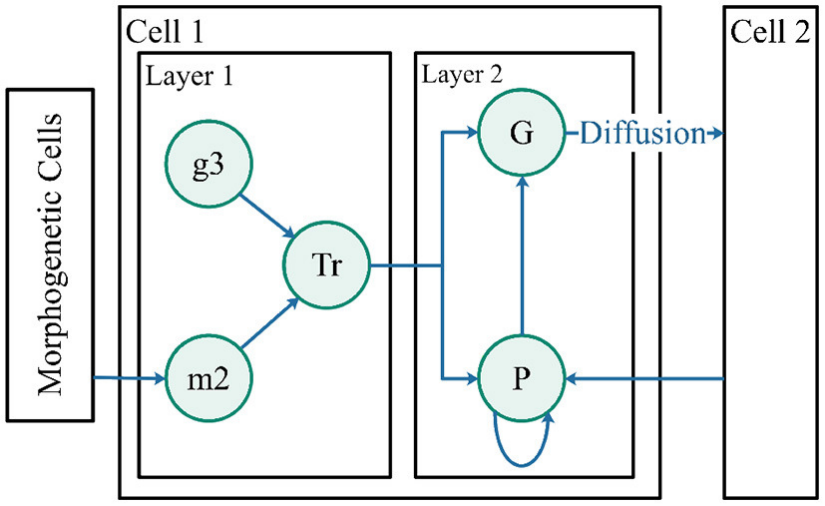
Robot control cells. The two layers represent the pattern generation and pattern formation. Morphogen m2 is derived from morphogenetic cells. Protein Tr is regulated by gene g3 and morphogen m2. Both proteins, G and P, are influenced by protein Tr.

Robot control cells represent robots, guiding robots to target in the pattern. A structure of double layers was designed for the robot control cells (Figure 6). The first layer is simplified into a single gene, g3, which is a random value for each robot within 0 to 1. The first layer of cells is the pattern interpretation layer used to receive morphogens from morphogenetic cells. The morphogenetic cell position where the in-cell concentration reaches the threshold can be used as a control point in “translating” protein Tr. Protein Tr translated the information into the NURBS model for input information in the second layer.

The second layer of cells adopts the control method described in this paper (Guo et al., 2011) to guide the robots to a suitable position in the pattern. The cell structure is shown in Figure 6.

*m*_2_ is the concentration of morphogens secreted by the morphogenetic cells. The morphogen concentrations in the morphogenetic cells activate gene *g*_3_, defined as list *P*_*mc*_(*i*)∈{*p*_*ix*_, *p*_*iy*_}. For n morphogenetic cells, *i* ∈ {1, *n*}, the activation equation is as follows:


(9) $$\begin{array}{l} g_{3i}=\{\begin{array}{cc} 1, & Sig\left(m_{2},\theta_{2},k_{2}\right)>\delta \\ 0, & others \end{array} \end{array}$$

For each cycle, if *g*_3_*i* = 1, the corresponding morphogenetic cell is ∈*P*_*mc*_. In this equation, the sigmoid formula is used as a non-linear activation to determine whether gene *g*_3_ containing the sub-genes is activated by input *m*_2_. *theta*_2_ and *k*_2_ are the parameters of the sigmoid function. Only the activated sub-genes are viewed as the control points.

Tr represents a protein that contains pattern information translated from *m*_2_. Tr first sorts *P*_*mc*_ using Algorithm 1, starting with the nearest morphogenetic cell in a clockwise direction of mode movement as the first point and searches for the nearest morphogenetic cell clockwise from the starting point as the next point, sorting in order.

**Algorithm 1:**
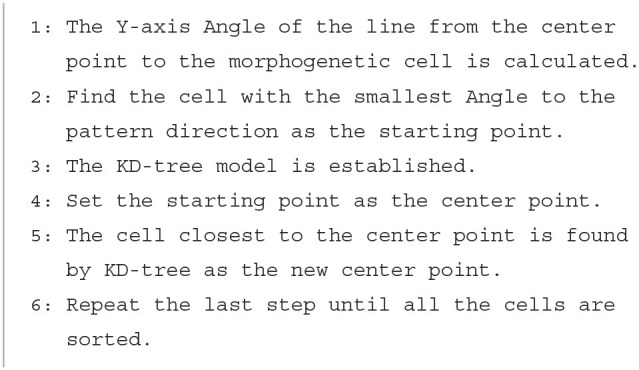
Translation algorithm.

The sorted points are taken as control points of the NURBS model to generate a pattern. The second layer of cells is the feedback control GRN layer. The first layer receives the updated morphogen from morphogenetic cells and translates it into the NURBS pattern, which serves as the second layer's input. The second layer is defined as follows:


(10) $$\begin{array}{l} \frac{dG_{i,x}}{dt}=-a{\text{z}}_{i,x}+mP_{i,x} \end{array}$$

(11) $$\begin{array}{l} \frac{dG_{i,y}}{dt}=-a{\text{z}}_{i,y}+mP_{i,y} \end{array}$$

(12) $$\begin{array}{l} \frac{dP_{i,x}}{dt}=-cP_{i,x}+rf\left({\text{z}}_{i,z}\right)+bD_{i,x} \end{array}$$

(13) $$\begin{array}{l} \frac{dP_{i,y}}{dt}=-cP_{i,y}+rf\left({\text{z}}_{i,z}\right)+bD_{i,y} \end{array}$$

where *i* = 1, 2, ..., *n* denotes all robots in the swarm. Protein *G* stores the location information, representing the coordinates of the robot in the local coordinate system, corresponding to *G*_*I,x*_ and *G*_*I,y*_. Protein *P* represents the internal state of the robots, with *P*_*I,x*_ and *P*_*I,y*_. When protein *G* diffuses into the extracellular space, its concentration state is expressed as *D*_*i*_. *D*_*i*_ denotes the sum of the vector forces between the current robot and its neighbors.


(14) $$\begin{array}{l} D_{i,x}=\overset{N_{i}}{\underset{j=1}{\sum }}\frac{\left(G_{i,x}-G_{j,x}\right)}{\sqrt{{\left(G_{i,x}-G_{j,x}\right)}^{2}+{\left(G_{i,y}-G_{j,y}\right)}^{2}}} \end{array}$$

(15) $$\begin{array}{l} D_{i,y}=\overset{N_{i}}{\underset{j=1}{\sum }}\frac{\left(G_{i,y}-G_{j,y}\right)}{\sqrt{{\left(G_{i,x}-G_{j,x}\right)}^{2}+{\left(G_{i,y}-G_{j,y}\right)}^{2}}} \end{array}$$

*N*_*i*_ denotes the number of robots in the perceived range of the ith robot.


(16) $$\begin{array}{l} f\left({\text{z}}_{i,x}\right)=\frac{1-e^{-{\text{z}}_{i,x}}}{1+e^{-{\text{z}}_{i,x}}} \end{array}$$

(17) $$\begin{array}{l} f\left({\text{z}}_{i,y}\right)=\frac{1-e^{-{\text{z}}_{i,y}}}{1+e^{-{\text{z}}_{i,y}}} \end{array}$$

In this equation, *z*_*I,x*_ and *z*_*I,y*_ are defined as the entrances of the second layer.


(18) $$\begin{array}{l} z_{i,x}=\left(G_{i,x}-NURBS_{i,x}\left(u\right)\right) \end{array}$$

(19) $$\begin{array}{l} z_{i,y}=\left(G_{i,y}-NURBS_{i,y}\left(u\right)\right) \end{array}$$

*NURBS*_*I,x*_(*u*) and *NURBS*_*I,y*_(*u*) denote the NURBS pattern translated by the first layer of protein Tr. The NURBS model accepts the input parameter *u*, and the coordinates of any point on the NURBS pattern can be obtained. The value range of u ranged from 0 to 1.

## 5. Results and discussions

**Figure 7:**
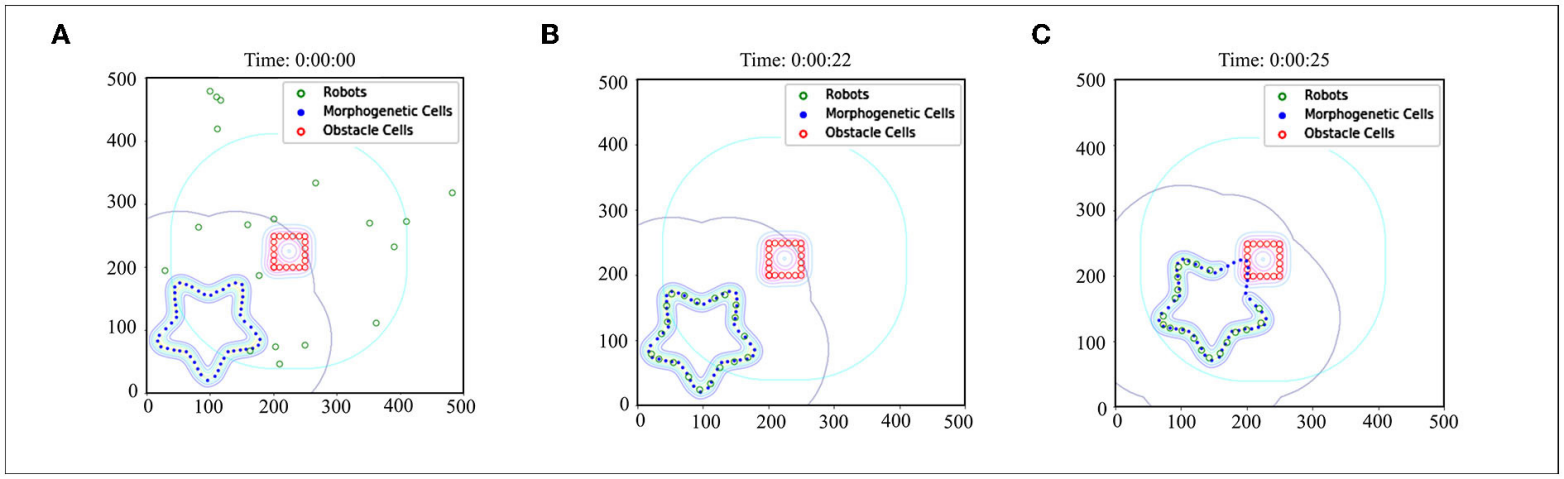
Case of CR-GRN. **(A)** Initial state: 20 robots were randomly distributed in the scene. **(B)** Robots were arranged in the target pattern. **(C)** The robots moved with the movement of morphogenetic cells, and the obstacle cells inhibited the activity of morphogenetic cells, which indirectly affected the distribution of robots in the scene.

A demo is used to demonstrate the basic effects of CR-GRN, as shown in Figure 7. The performance of the CR-GRN model was systematically evaluated. First, we compared the CR-GRN model with the H-GRN model. The CR-GRN model's performance was evaluated in the pattern formation process, movement stability, interaction with obstacles, etc. In the simulation, each green circle represents a robot, the blue circle represents morphogenetic cells, and the red circle represents obstacles. The maximum movement speed of the robots was 20 cm/s. Each control cycle was 0.1.

### 5.1. Ability of generating pattern formations

To test the pattern formation performance of our proposed model, we used a 500 × 500 cm experimental scenario in which multiple robots worked together to complete the pattern formation. The robot's maximum moving speed was 20 cm/s.

The predefined patterns required were provided by pictures. After binarization and skeletonization, the skeletonization area points were extracted, and 200 points were randomly selected as control points to generate a pattern represented by the NURBS model. For NURBS to generate a continuous curve along the sequence of control points, we sort the 200 points by the KD-tree, with the upper left corner point as the starting and ending points and the remaining points arranged in order of distance. Furthermore, the number of morphogenic cells was set as 50, and each morphogenic cell was assigned a value of 0 to 1, through which coordinates on the corresponding NURBS pattern were obtained.

A series of target patterns was chosen from simple to complex, and a total of 10, 36, and 49 robots were used to produce patterns. Table 1 shows the time spent in forming the formations for different patterns and different numbers of robots, and the trend is shown in Figure 5C.

**Table 1:** Time taken to form a formation.

**Pattern names**	**No. of robots**		
	**10**	**36**	**49**
Pattern1	9.696 ± 0.765	24.892 ± 2.011	60.676 ± 15.887
Pattern2	9.428 ± 1.037	28.296 ± 2.823	61.784 ± 9.164
Pattern3	9.552 ± 0.736	25.072 ± 2.940	56.248 ± 15.005
Pattern4	9.524 ± 0.859	26.240 ± 2.554	60.600 ± 12.301

To quantify the pattern formation performance, a series of simulations were conducted to evaluate the average position error, that is, the average minimum distance between the robots and the target pattern. The result is shown in the Table 2. If the deviation was greater than 10 within 100 s, the robots were considered to have failed to complete the task, and the error value was the average number of robots that failed to complete the task.

**Table 2:** Mean deviation in forming the pattern formation.

**Pattern names**	**No. of robots**		
	**10**	**36**	**49**
Pattern1	0.410 ± 0.357	1.031 ± 0.298	1.109 ± 0.348
Pattern2	0.562 ± 0.395	1.186 ± 0.531	1.293 ± 0.480
Pattern3	0.762 ± 0.565	1.271 ± 1.122	1.415 ± 0.502
Pattern4	0.705 ± 0.888	1.162 ± 1.273	1.440 ± 1.240
Errors	0	0	10.680

### 5.2. Ability of maintaining pattern

**Figure 8:**
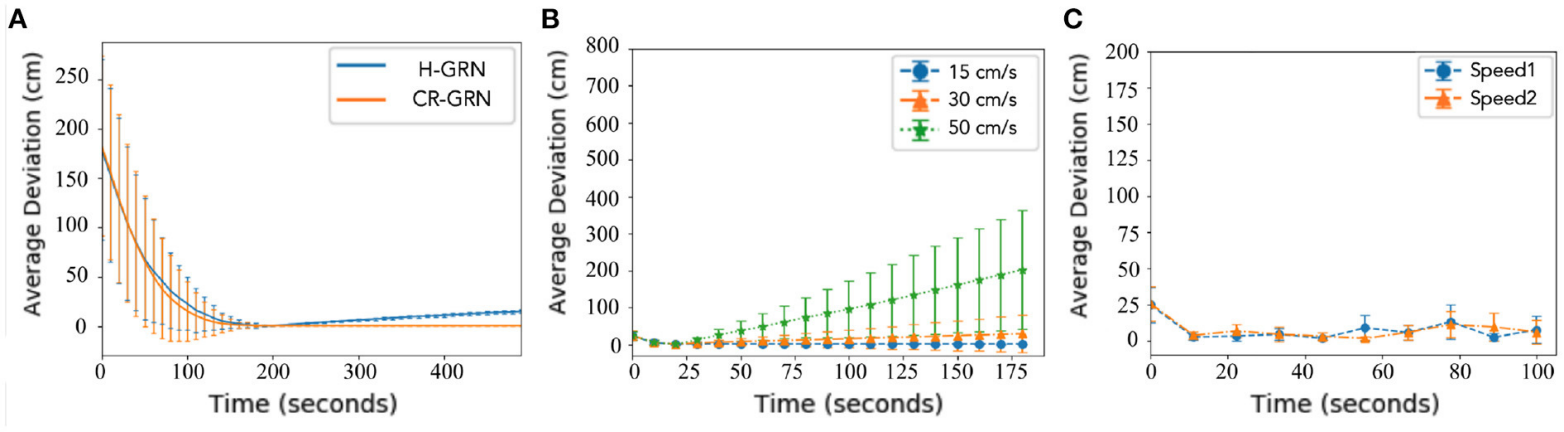
Stability. The Average shortest distance to pattern between robots and pattern are calculated as average deviation. **(A)** H-GRN and CR-GRN were compared for the trapping target task. **(B)** Straight movement and **(C)** curved movement (V1 = 5 cm/s, R1 = 0.052 rad/s, V2 = 10 cm/s, R2 = 0.087 rad/s, and robot no. = 20) was performed using the CR-GRN.

In this section, the robots' stability to maintain pattern formation is discussed.For comparison, the H-GRN algorithm was selected as a reference. The number of robots set in the simulation experiment is 20. A 500 × 500 cm simulation scenario is used for multiple robots worked together to complete the pattern formation. The robot's maximum moving speed was 20 cm/s. In addition, for this purpose, the two methods required the same criteria because the H-GRN was not designed for the predefined pattern. Both algorithms were set as tasks for trapping targets. A tested scenario was designed in which the robots needed to trap a different number of targets. Then, the average deviations between robots and the expected pattern were observed. The results are shown in Figure 8A. We calculate the shortest distance from each robot to the target pattern. It is assumed that the smaller the average shortest distance, the better the robot can maintain the pattern formation. We also calculated the standard deviation of the shortest distance. The smaller the standard deviation, the fewer outliers. The results show that the CR-GRN can achieve formation faster than the H-GRN and maintain formation more easily over time.

### 5.3. Ability of formation movement in maneuver

The most critical question in this study is maintaining the formation in the maneuver. In this section, the algorithm's ability to maintain maneuver formation is discussed. The maintenance of formations is divided into two different situations. (1) Fast movement and slow movement, (2) straight movement and curved movement.

**Figure 9:**
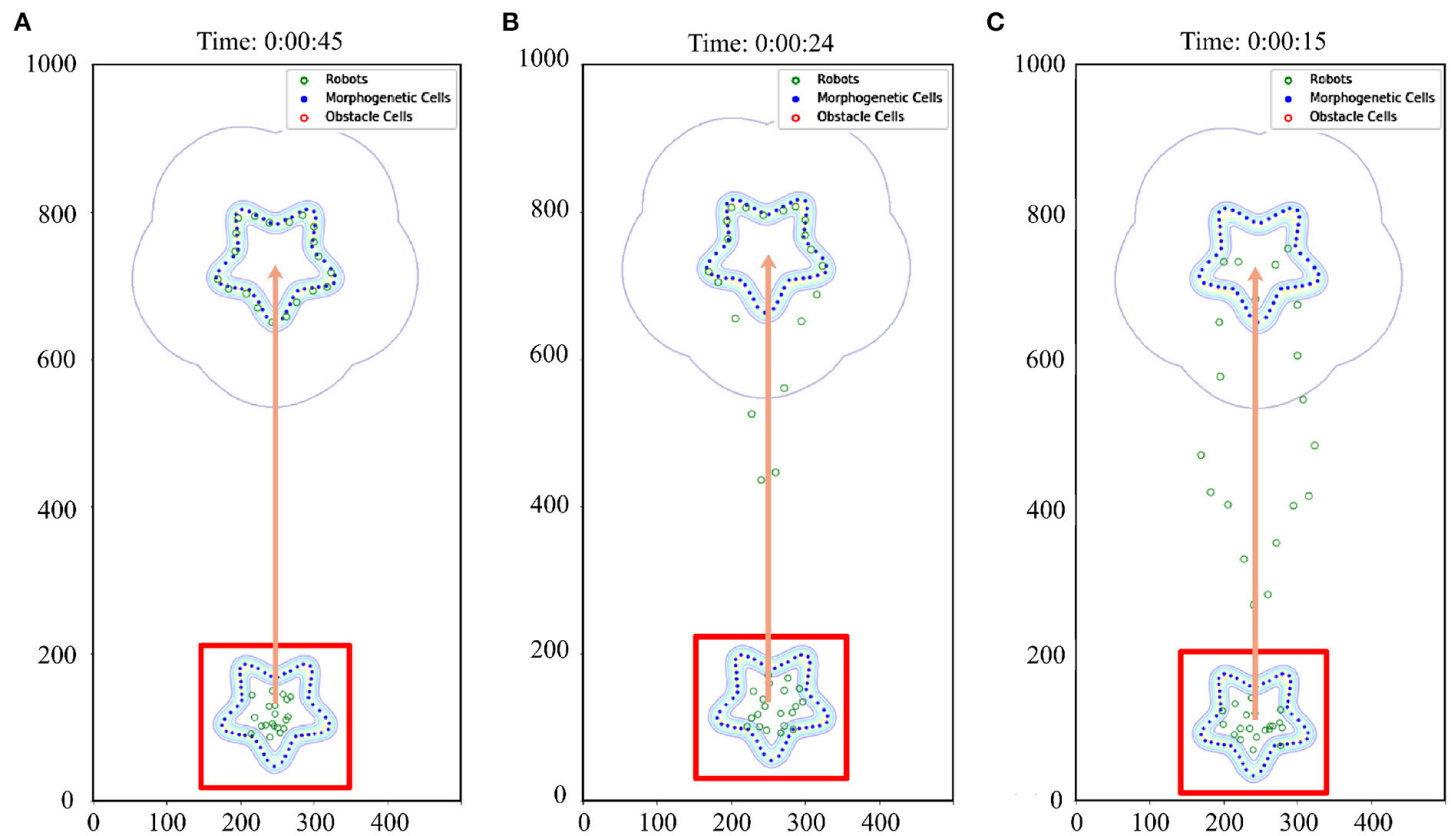
Effect of different speeds on formation. The red box indicates the initial state. When the morphogenetic cells moved forward, the robot followed the target. The orange arrow indicates the direction of movement of the robot formation. **(A)** V = 15 cm/s, **(B)** V = 30 cm/s, **(C)** V = 45 cm/s.

The number of robots set in the simulation experiment is 20. A 500 × 1,000 cm simulation scenario is set. The robot's maximum moving speed was 20 cm/s. By setting the speed (15, 30, 45 cm/s) at which morphogenetic cells guide the robot's movement, we tested the swarm behavior of the robot. The simulation results are shown in Figure 9, where the movement states are depicted at three different speeds. We recorded the robot's average error at different speeds and at different times, as shown in Figure 8B. The trajectory recording is presented in **Figure 11A**.

**Figure 10:**
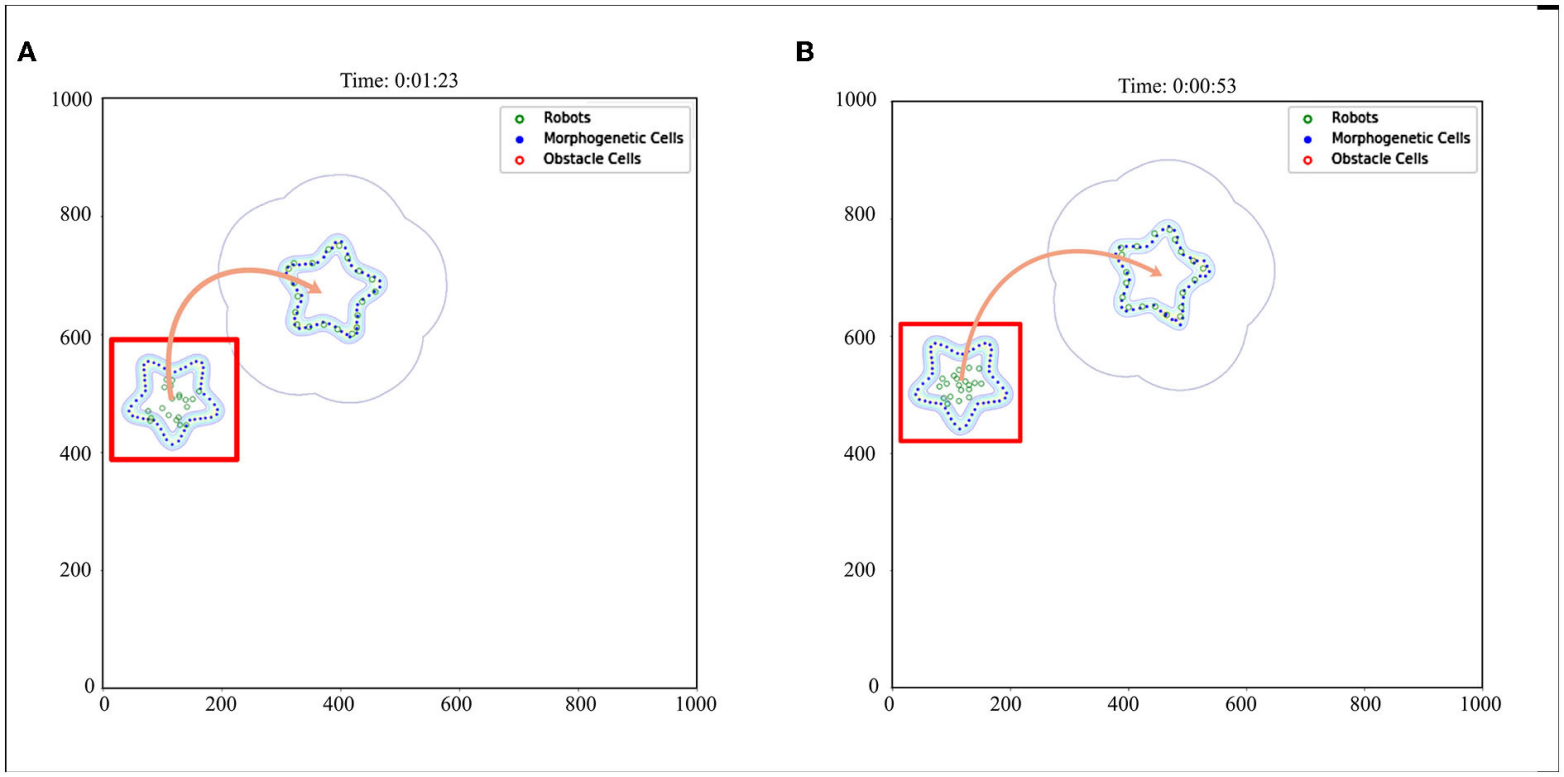
Curve movement. The red box indicates the initial state. The red box indicates the initial state. The orange arrow indicates the direction of movement of the robot formation. When the morphogenetic cells moved forward, the robot followed the target. **(A)** V = 5 cm/s, R = 0.052 rad/s, and **(B)** V = 10 cm/s, R = 0.087 rad/s.

**Figure 11:**
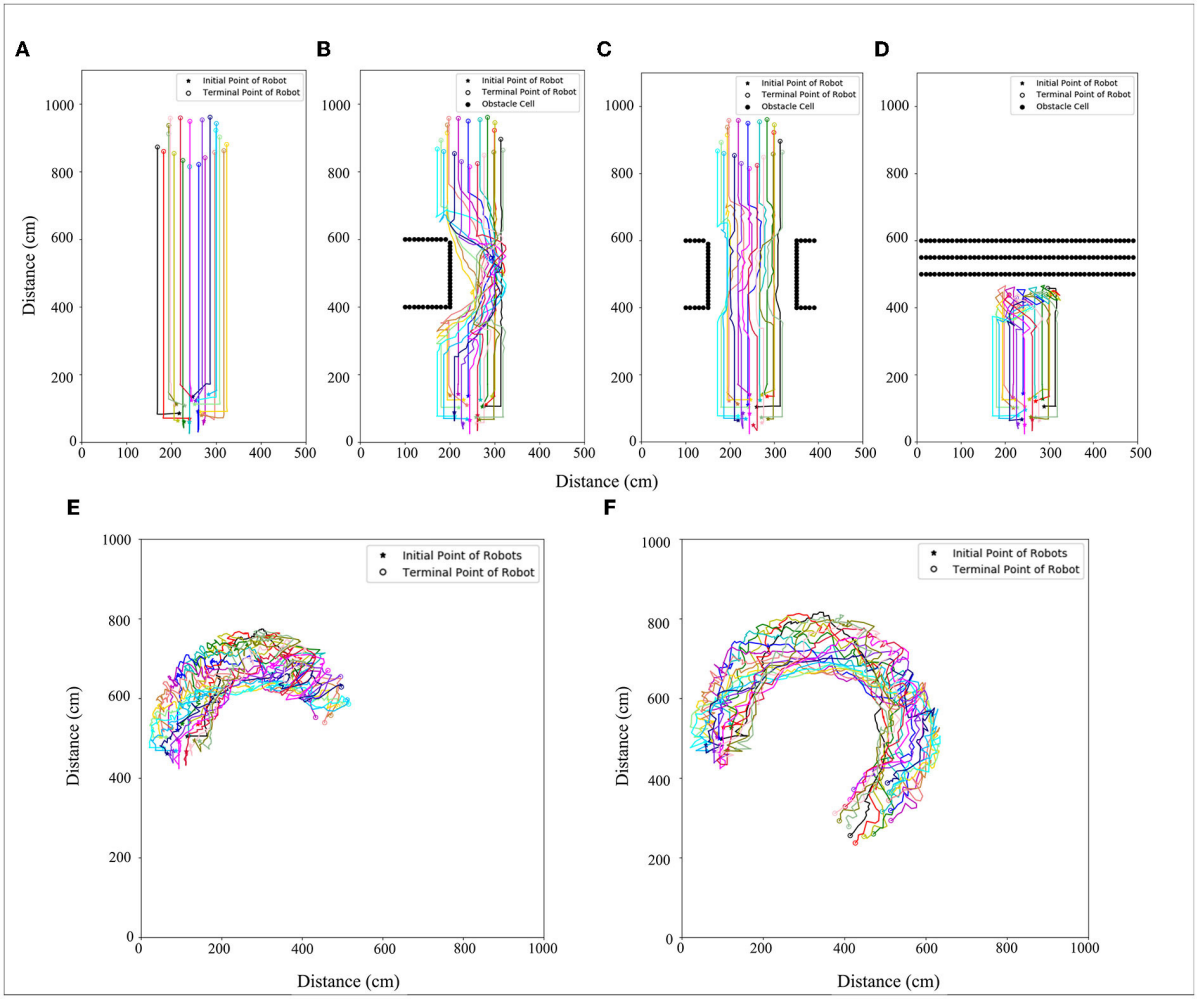
Trajectory with **(A)** no obstacles, **(B)** obstacle type 1, **(C)** obstacle type 2, **(D)** obstacle type 3, and **(E)** curved movement (V = 5 cm/s, R = 0.052 rad/s), **(F)** curved movement (V = 10 cm/s R = 0.087 rad/s). Different color lines are used to represent different robots trajectories.

We also established a test scheme for curved movement that changed the morphogenetic cell angle curve. And a larger experimental scenario (1,000 × 1,000) is used for simulation. The simulation results are shown in Figure 10. Corresponding trajectory recording is shown in Figures 11E,F Although the curve trajectory was complex, the average error exhibited good formation maintenance, as shown in Figure 8C.

### 5.4. Adaptability of model to the environment

**Figure 12:**
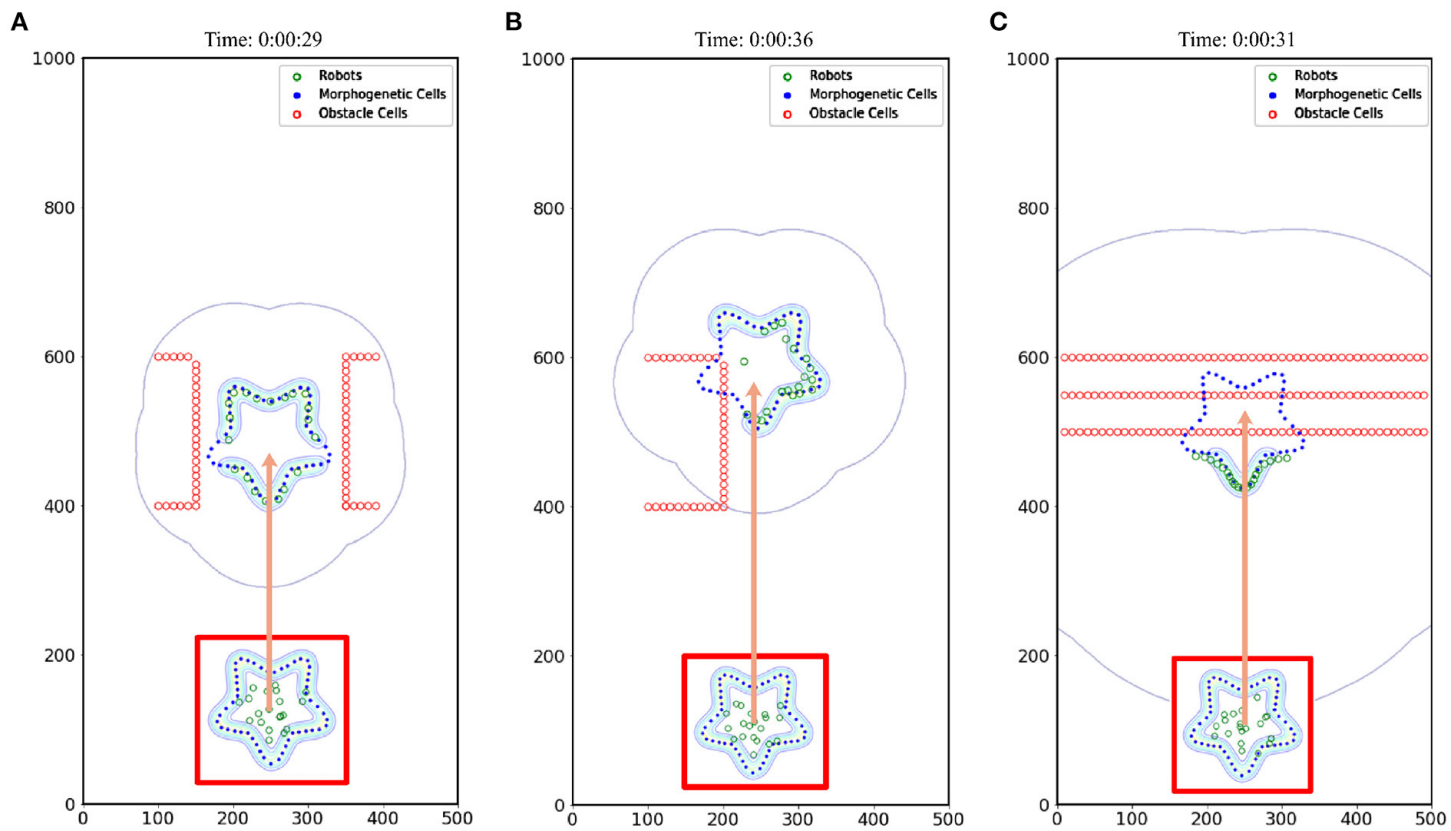
Obstacle avoidance. **(A)** Obstacle type 1, **(B)** Obstacle type 2, and **(C)** Obstacle type 3.

This section details how the robots interact with the environment and the adaptive changes. To cope with this problem, we made the robots pass through the barrier area and evaluated the pattern shape change. The surfaces of the obstacles were separated into obstacle cells. The simulation results are shown in Figure 12, and the corresponding trajectory changes are shown in Figures 11B–D.

### 5.5. Discussion

The simulation results are analyzed and discussed in this section. First, given the ability of the CR-GRN in generating pattern of the proposed algorithm, it is shown that the robots can complete formation in time under the guidance of morphogens by observing the simulation results. This method makes the resulting pattern more stable by applying the proposed static diffusion equation. The algorithm has high stability and response speed in movement. Whether it is straight or curved, the movement of morphogenetic cells can be fast followed by robots without significant deviation from the target formation. In terms of adaptability, robots cross obstacles by deforming patterns created by morphogenetic cells that interact with the environment. The simulation results show that the algorithm has good pattern formation ability, high pattern stability, stable formation during maneuvering, and good interaction with the environment. This algorithm provides a new and effective solution for pattern formation maneuver problems.

## 6. Conclusion and future directions

In this paper, we propose the CR-GRN to manage maneuver control of adaptive pattern formation. To maintain stable patterns in dynamic environments, a static diffusion function is proposed. Then, the cell reaction network is introduced for the interaction between the robots and the environment by pattern construction. Combined with the proposed morphogen static diffusion equation and the principles of the GRN method, the cellular reaction network can inspire us to study the GRN from a new perspective. Some simulation experiments are designed and conducted to validate the effects of the proposed method, and the results show good performance in maneuvering pattern formation. However, the static diffusion equation can calculate the concentration of morphogens well, and there are some limitations that cannot be directly applied to the reaction-diffusion problem when multi-morphogens exist. Extending this equation to the reaction-diffusion problem will further promote the study of the morphogenetic algorithm. The introduction of a cellular reaction network is not only beneficial for solving non-linear problems but also makes the system more complex. The movement control layer allows robots to be evenly distributed in a pattern, which is unfriendly to the scalability of swarm systems and relies too much on parameter settings. In some rare cases, robots will be unable to avoid obstacles when the formation passes through them. And the algorithm does not provide collision avoidance ability between robots in the process of motion. Future work can be focused on the scalability and stability of the swarm by improving the motion control layer. And the applicability of the algorithm will be further studied, such as the collision avoidance mechanism between robots in motion.

## Data availability statement

The raw data supporting the conclusions of this article will be made available by the authors, without undue reservation.

## Author contributions

ZX contributed to conception and design of the study, and wrote the first draft of the manuscript. All authors contributed to manuscript revision, read, and approved the submitted version.

## Funding

This work was supported in part by the Joint Funds of the National Natural Science Foundation of China (No. U1913206) and Shenzhen Science and Technology Program (No. JSGG20211029095205007 and No.JSGG20200701095404008).

## Conflict of interest

The authors declare that the research was conducted in the absence of any commercial or financial relationships that could be construed as a potential conflict of interest.

## Publisher's note

All claims expressed in this article are solely those of the authors and do not necessarily represent those of their affiliated organizations, or those of the publisher, the editors and the reviewers. Any product that may be evaluated in this article, or claim that may be made by its manufacturer, is not guaranteed or endorsed by the publisher.
